# Novel Disease-Specific Panel of Salivary microRNAs for the Detection of Oral Squamous Cell Carcinoma from Early Invasion to Stage IV Disease

**DOI:** 10.3390/ijms27115138

**Published:** 2026-06-05

**Authors:** Iphigenia Gintoni, Stavros Vassiliou, Myrto Kardara Bellou, Athanasios Balakas, Nikolaos Lefantzis, Veronica Papakosta, Dimitrios Vlachakis, George P. Chrousos, Christos Yapijakis

**Affiliations:** 1 Unit of Orofacial Genetics, 1st Department of Pediatrics, School of Medicine, National Kapodistrian University of Athens, “Aghia Sophia” Children’s Hospital, 115 27 Athens, Greece; 2 Choremion Laboratory, University Research Institute of Maternal and Child Health and Precision Medicine, “Aghia Sophia” Children’s Hospital, 115 27 Athens, Greece; dimvl@aua.gr (D.V.); chrousge@med.uoa.gr (G.P.C.); 3 Department of Oral and Maxillofacial Surgery, School of Medicine, National Kapodistrian University of Athens, Attikon Hospital, 124 62 Athens, Greece; stvasil@med.uoa.gr (S.V.); abalakas63@gmail.com (A.B.); vpapakosta71@gmail.com (V.P.); 4 Laboratory of Genetics, Department of Biotechnology, School of Applied Biology and Biotechnology, Agricultural University of Athens, 11855 Athens, Greece

**Keywords:** oral cancer, OSCC, early invasion, microRNA, miR-20b-5p, miR-181d-5p, miR-185-5p, miR-484, biomarker, saliva, liquid biopsy, early diagnosis, presymptomatic detection

## Abstract

Oral squamous cell carcinoma (OSCC) is characterized by consistently high mortality rates (≤60%) despite therapeutic advances. This is attributable to diagnostic delays arising from the asymptomatic early stages and time-consuming protocols. Hence, the establishment of reliable biomarkers for the routine assessment of the oral mucosa is imperative. MicroRNAs (miRNAs), key epigenetic regulators of gene expression, represent ideal candidates given their characteristic dysregulation across different pathologies. Here, we aimed to identify novel OSCC-specific miRNAs for the saliva-based detection of OSCC from the presymptomatic stage of early invasion. Through a multistep bioinformatic workflow, four miRNAs (miR-20b-5p, miR-484, miR-185-5p and miR-181d-5p) were identified as disease-specific since they simultaneously regulated >65% of a panel encompassing the 15 primarily overexpressed oncogenes in OSCC and a stage-specific panel including the six upregulated genes that genetically define the malignant stages of sequential oral carcinogenesis. The salivary expression of the identified miRNAs was studied in 31 OSCC patients and 31 healthy controls, using quantitative real-time PCR, followed by statistical analysis and an evaluation of the diagnostic accuracy. All studied miRNAs were significantly downregulated in the saliva of OSCC patients compared to controls (miR-484, *p* < 0.001; miR-181d-5p, *p* < 0.001; miR-185, *p* = 0.008; miR-20b, *p* = 0.026) and exhibited combinatory diagnostic performance of 95.4% (*p* < 0.001) for OSCC detection. Their expression remained uninfluenced by lifestyle and clinicopathological parameters, including smoking/alcohol, tumor site, grade and disease stage. The proposed 4-miRNA panel exhibits high diagnostic performance for the early, saliva-based detection of OSCC, irrespective of histopathological and lifestyle confounders, highlighting its potential as a robust non-invasive screening tool.

## 1. Introduction

Oral cancer represents the sixth most prevalent malignancy worldwide, with more than 1.5 million cases reported only in 2021 and an estimate of approximately 83% increased incidence by 2050 [1,2]. Over 90% of oral cancer cases are represented by oral squamous cell carcinoma (OSCC), which clinically manifests as exophytic lesions or ulcers of the oral cavity [3,4], affecting multiple sites of the maxillofacial region, including the tongue, buccal and alveolar mucosae, gingiva and hard and soft palates, as well as the floor of the mouth and lips [5,6,7]. OSCC predominantly affects individuals between 50 and 70 years of age, with a marked male predominance [8]. Smoking and alcohol consumption constitute the two principal risk factors associated with OSCC development globally, while viral infections and poor oral hygiene, as well as chronic inflammation or repetitive trauma of the oral epithelium, further contribute to its pathogenesis [3,5,9]. OSCC is characterized by outstandingly high aggressiveness, associated with elevated rates of lymph node involvement and metastases, affecting 40–60% of patients, as well as recurrence in approximately 30% of post-treatment cases [10,11].

Patients frequently encounter profound functional and esthetic deficits, which significantly affect quality of life. Related morbidity may involve intensive pain; bleeding; impairments in mastication, swallowing or speech; and compromised flavor perception, as well as severe maxillofacial deformities resulting from the invasion of adjacent tissues and bone structures [12,13]. Standard surgical treatment of OSCC tumors, as well as chemotherapy and radiotherapy, is not universally effective and has been shown to cause further deterioration in patients’ wellbeing, leading to a series of chronic complications, such as permanent disfigurement, osteonecrosis, exposed bone structures, loss of teeth and salivary gland dysfunction [7,14,15,16].

The mortality related to OSCC is considered the highest among all head and neck neoplasms, reaching up to 60% within the first 5 years and thus representing a significant global burden [17,18,19]. Survival rates among OSCC patients have remained unchanged for decades and, conversely, demonstrate an annual declining trend despite significant advances in treatment and histopathological assessment [14,20,21,22,23]. Besides the aggressive nature of OSCC, the persistently poor prognostic outcomes of affected patients are primarily attributable to extensive diagnostic delays. These are further compounded by time-consuming protocols, as more than 50% of patients present with stage III or stage IV disease, when the neoplasm has already infiltrated the surrounding tissues or has metastasized to the regional lymph nodes and, in certain cases, to the distant organs [9,16,17,24]. The disease stage at diagnosis constitutes a key prognostic indicator, as the mortality rates accompanying the detection of late-stage OSCC can be as high as 70%, compared to 20% at stages I and II [5,25]. The delayed clinical presentation of OSCC is largely attributable to the asymptomatic nature of early tumorigenesis in the majority of cases [9,16]. In contrast, OSCC patients with precursor premalignant lesions, such as leukoplakia, that are routinely monitored and undergo malignant transformation over time, face significantly greater 5-year survival odds (around 80%), as their diagnosis predominantly takes place at stage I [26,27].

The histopathological diagnosis of OSCC is only applicable to clinically apparent lesions that have driven patients to seek medical attention. Hence, the establishment of protocols for the routine assessment of the oral mucosa is deemed critical in order to enable the early detection of carcinogenesis and ultimately shift prognosis and quality of life towards more favorable outcomes [9,17,22,28]. Given its direct contact with the site of tumorigenesis, saliva represents the most reliable biofluid for the non-invasive diagnosis of OSSC, and its simple, atraumatic collection allows for regular monitoring of the oral epithelium [29,30]. However, the clinical utility of saliva as a diagnostic tool requires the identification of sensitive biomarkers specific to OSCC, which have not been established so far [9,17,29,31].

Among the molecular candidates for the non-invasive detection of OSCC, microRNAs (miRNAs), a class of endogenous, small non-coding RNAs, stand out as particularly compelling [29,32,33]. This is attributed to their established role as epigenetic regulators of gene expression and their robust biofluid stability, as well as their characteristic and quantifiable expression patterns across different pathologies, including OSCC [9,31,33]. miRNAs negatively regulate the expression of their target genes by binding to the 3′ untranslated regions (3′ UTRs) of their mature mRNAs. 3′ UTR recognition is mediated by the seed region of the 5′ end of the miRNA (nucleotides 2–7) and results in post-transcriptional repression, depending on the degree of target complementarity (partial or complete) [19,34].

Building upon the well-characterized genetic landscape of OSCC [18,35,36], we have recently developed two integrative bioinformatic models for the identification of the most specific miRNAs for OSCC, within a two-tier framework [18,25]. The first model was developed to capture the most disease-specific miRNAs that simultaneously regulate the expression of the majority of OSCC driver genes while demonstrating the reverse pattern of dysregulated expression in disease-related biospecimens [18]. The second, award-winning, complementary model [25] enabled the corresponding identification of miRNAs specific to each stage of oral carcinogenesis by incorporating their genetic signatures, according to our group’s distinguished in vivo model of sequential oral oncogenesis in Syrian hamsters [36,37]. Among the results of the stage-specific model, miR-34a-5p was identified as a potential biphasic biomarker with distinct expression patterns between the premalignant and malignant stages of OSCC progression. This bioinformatic prediction was later validated by a proof-of-concept experimental study that revealed its state-dependent dysregulation across normal mucosae, premalignant lesions and OSCC (*p* < 0.001) [9].

In the present bioinformatic and experimental study, we aimed to identify novel OSCC-specific miRNAs with diagnostic potential for the reliable saliva-based detection of OSCC, from the earliest presymptomatic stage of early invasion, by leveraging the complementary strengths of our two models in a combinatorial approach.

## 2. Results

### 2.1. Bioinformatic Identification of OSCC-Specific miRNAs from the Stage of Early Invasion

**Figure 1:**
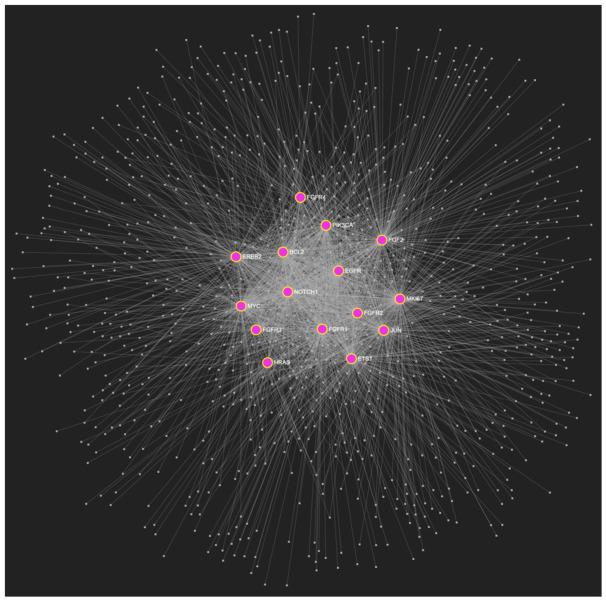
miRNA/target interaction network depicting the 1034 miRNA molecules regulating the post-transcriptional expression of at least one of the main 15 driver oncogenes that comprise the disease-specific panel developed for OSCC [18]. The miRNAs are illustrated as gray dots at the ends of connecting nodes across the network, while the encompassed upregulated oncogenes of the panel are illustrated as pink circular elements and include *EGFR*, *NOTCH1*, *PIK3CA*, *JUN*, *MYC*, *HRAS*, *ERBB2*, *FGFR4*, *ETS1*, *BCL2*, *MKI67*, *FGF2*, *FGFR1*, *FGFR2* and *FGFR3*.

**Figure 2:**
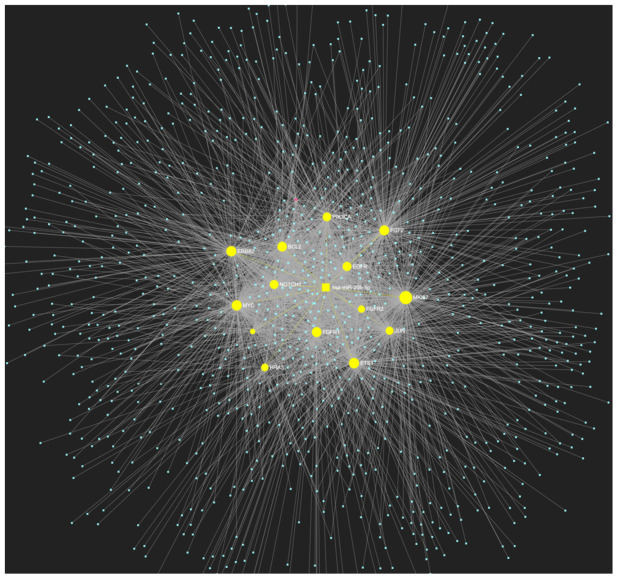
miRNA/target interaction network depicting the shared interactions of miR-20b-5p, which simultaneously regulates 14/15 oncogenes of the disease-specific panel (93.3%), except *FGFR4*. miR-20b-5p is illustrated as the central yellow square, while the genes that are simultaneously regulated by the miRNA are represented as yellow circular elements, along with their corresponding nodes. The light blue elements/nodes correspond to the remaining miRNAs of the initial network, targeting at least one included oncogene of the panel, while the gene not targeted by miR-20b-5p is illustrated in pink.

**Figure 3:**
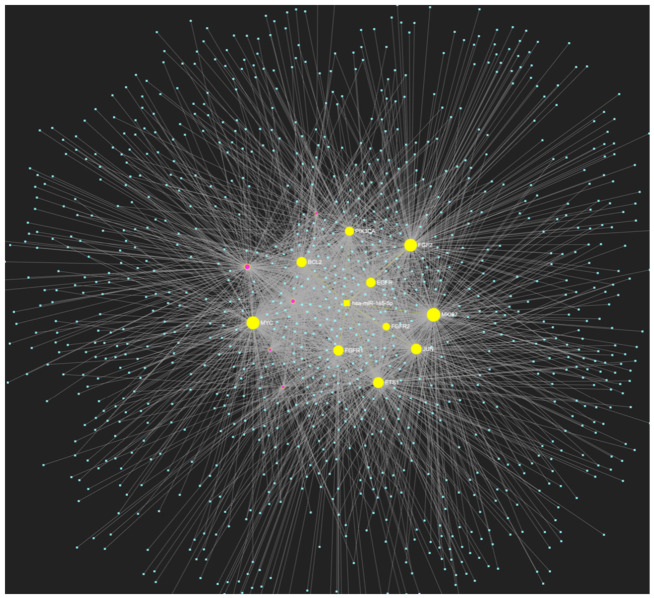
miRNA/target interaction network depicting the shared interactions of miR-185-5p, which simultaneously regulates 10/15 oncogenes of the disease-specific panel (66.7%), except *NOTCH1*, *ERBB2*, *HRAS*, *FGFR3* and *FGFR4*. miR-185-5p is illustrated as the central yellow square, while the genes that are simultaneously regulated by the miRNA are represented as yellow circular elements, along with their corresponding nodes. The light blue elements/nodes correspond to the remaining miRNAs of the initial network, targeting at least one included oncogene of the panel, while genes not targeted by miR-185-5p are illustrated in pink.

**Figure 4:**
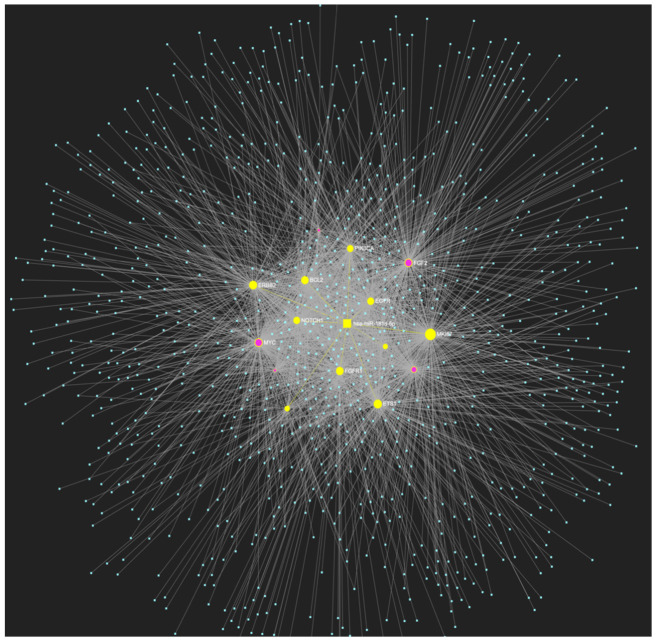
miRNA/target interaction network depicting the shared interactions of miR-181d-5p, which simultaneously regulates 10/15 oncogenes of the disease-specific panel (66.7%), except *JUN*, *MYC*, *FGF2*, *FGFR3* and *FGFR4*. miR-181d-5p is illustrated as the central yellow square, while the genes that are simultaneously regulated by the miRNA are represented as yellow circular elements, along with their corresponding nodes. The light blue elements/nodes correspond to the remaining miRNAs of the initial network, targeting at least one included oncogene of the panel, while genes not targeted by miR-181d-5p are illustrated in pink.

At a disease level, from the initial pool of 1034 candidates (Figure 1), the multistep bioinformatic strategy led to the identification of three miRNAs (miR-20b-5p, miR-185-5p and miR-181d-5p) that simultaneously regulated 66.6–93.3% of the oncogenes in the developed disease-specific panel (*EGFR*, *NOTCH1*, *PIK3CA*, *JUN*, *MYC*, *HRAS*, *ERBB2*, *ETS1*, *BCL2*, *MKI67*, *FGF2*, *FGFR1*, *FGFR2*, *FGFR3* and *FGFR4*) (Table 1) based on available direct experimental evidence focused on epithelial cells of the digestive tract and/or malignant phenotypes and with no previously reported expression patterns in OSCC-related specimens (Figure 2, Figure 3 and Figure 4).

**Figure 5:**
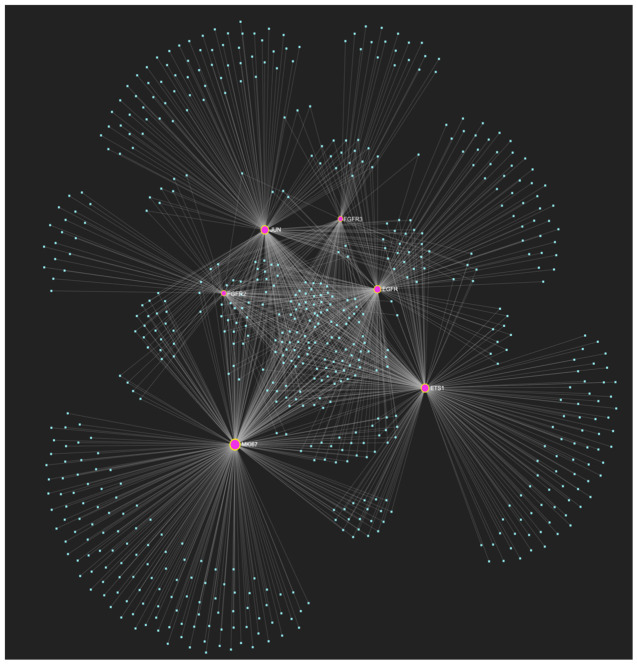
miRNA/target interaction network depicting the 635 miRNA molecules regulating the post-transcriptional expression of at least one of the 6 upregulated genes (*EGFR*, *JUN*, *ETS1*, *MKI67*, *FGFR2*, *FGFR3*) during the sequential malignant stages of oral oncogenesis (early invasion, well differentiated and moderately differentiated OSCC) [25]. The miRNAs are illustrated as light blue dots at the ends of connecting nodes across the network, while the encompassed upregulated oncogenes of the panel are illustrated as pink circular elements.

**Figure 6:**
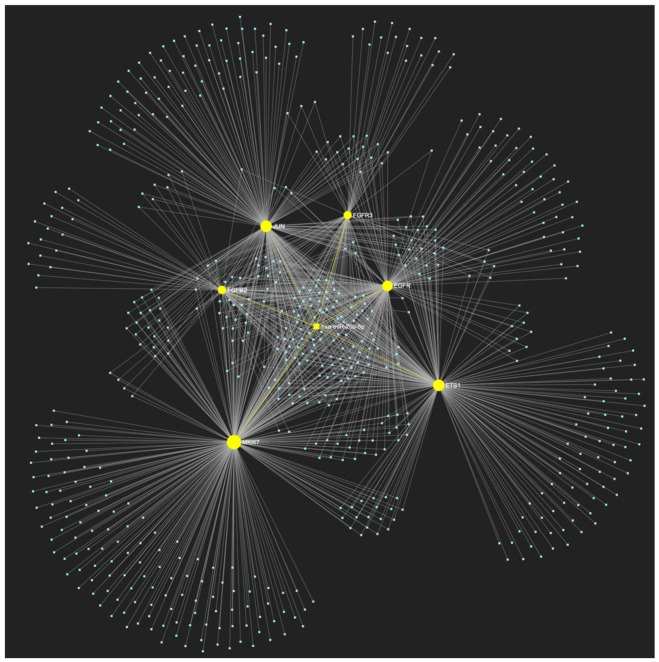
miRNA/target interaction network depicting the shared interactions of miR-20b-5p, which simultaneously regulates all six upregulated genes of the stage-specific panel (100.0%). miR-20b-5p is illustrated as the central yellow square, while the genes that are simultaneously regulated by the miRNA are represented as yellow circular elements, along with their corresponding nodes. The light blue elements/nodes correspond to the remaining miRNAs of the initial network, targeting at least one included gene of the panel.

**Figure 7:**
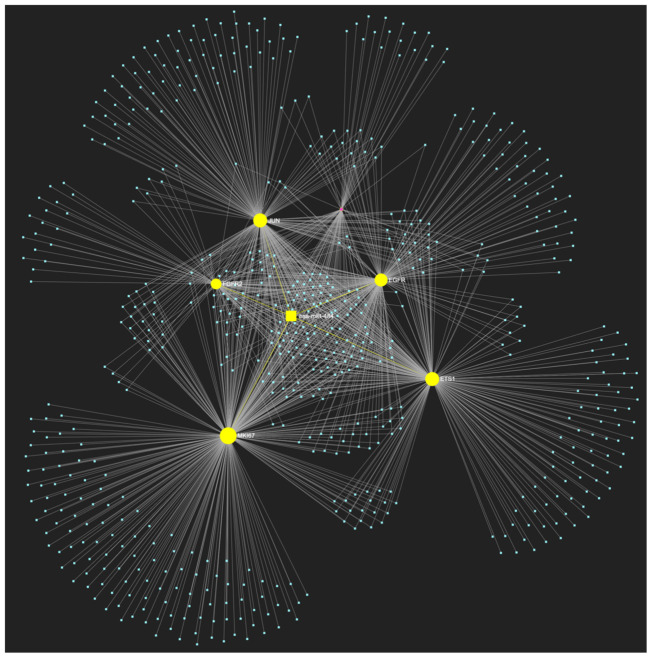
miRNA/target interaction network depicting the shared interactions of miR-484, which simultaneously regulates 5/6 upregulated genes of the stage-specific panel (83.3%), except *FGFR3*. miR-484 is illustrated as the central yellow square, while the genes that are simultaneously regulated by the miRNA are represented as yellow circular elements, along with their corresponding nodes. The light blue elements/nodes correspond to the remaining miRNAs of the initial network, targeting at least one included gene of the panel, while the gene not targeted by miR-484 is illustrated in pink.

**Figure 8:**
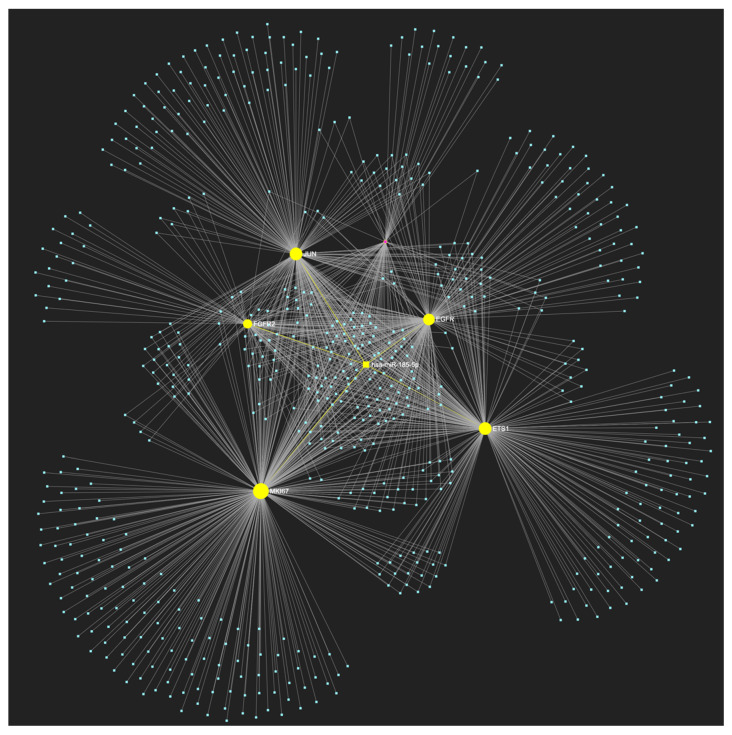
miRNA/target interaction network depicting the shared interactions of miR-185-5p, which simultaneously regulates 5/6 upregulated genes of the stage-specific panel (83.3%), except *FGFR3*. miR-185-5p is illustrated as the central yellow square, while the genes that are simultaneously regulated by the miRNA are represented as yellow circular elements, along with their corresponding nodes. The light blue elements/nodes correspond to the remaining miRNAs of the initial network, targeting at least one included gene of the panel, while the gene not targeted by miR-185-5p is illustrated in pink.

**Figure 9:**
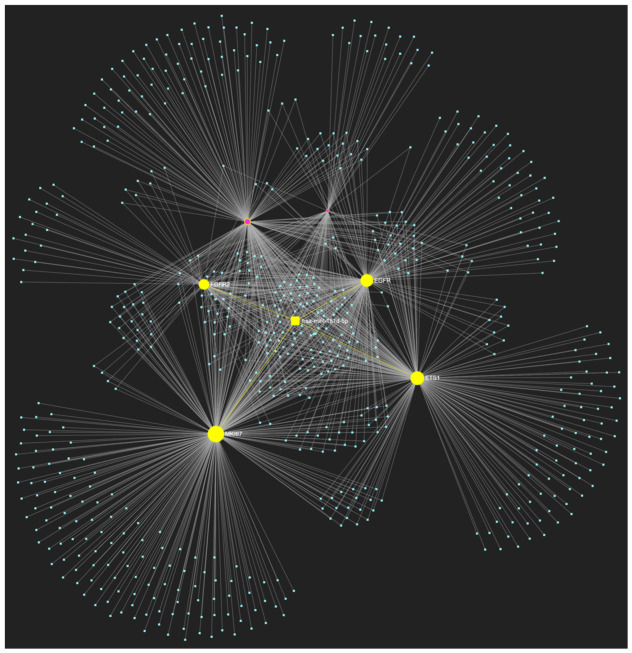
miRNA/target interaction network depicting the shared interactions of miR-181d-5p, which simultaneously regulates 4/6 upregulated genes of the stage-specific panel (66.7%), except *JUN* and *FGFR3*. miR-181d-5p is illustrated as the central yellow square, while the genes that are simultaneously regulated by the miRNA are represented as yellow circular elements, along with their corresponding nodes. The light blue elements/nodes correspond to the remaining miRNAs of the initial network, targeting at least one included gene of the panel, while genes not targeted by miR-181d-5p are illustrated in pink.

At a stage-specific level, the corresponding multistep bioinformatic analysis based on a customized panel (*EGFR*, *JUN*, *ETS1*, *MKI67*, *FGFR2*, *FGFR3*) comprising the upregulated genes during the sequential malignant stages of oral oncogenesis (early invasion, well differentiated and moderately differentiated OSCC), as defined by the representative hamster model of oral carcinogenesis, revealed four miRNAs among the 635 candidate molecules: miR-20b-5p, miR-484, miR-185-5p, miR-181d-5p. Among the identified miRNAs, which targeted 66.6–100% of the gene panel (Table 2; Figure 5, Figure 6, Figure 7, Figure 8 and Figure 9), three were common to the initial disease-level analysis. miR-484 (Figure 7), which emerged exclusively from the stage-specific analysis, was associated with 7 out of the 15 genes included in the customized panel of the disease-level model (*EGFR*, *NOTCH1*, *JUN*, *ERBB2*, *ETS1*, *MKI6* and *FGFR2*).

### 2.2. Demographic, Lifestyle and Clinicopathological Profiling of the Studied Groups

A total of 62 participants were included in the study, comprising 31 patients with OSCC and 31 healthy controls of comparable age and gender. Table 3 provides a summary of the demographic characteristics (age/gender) of all participants, as well as of the documented presence of OSCC-related lifestyle risk factors, such as smoking and alcohol consumption. The latter were further classified into light, moderate and heavy status, based on self-reported weekly cigarette packs or the intake of alcoholic beverages, using predefined thresholds. Light, moderate and heavy smoking were defined as <3, 3–7 and >7 packs per week, while the same categorization scheme for alcohol consumption included <3, 3–11.5 and >11.5 beverages per week, respectively.

Age was compared between OSCC patients and controls via the performance of an independent-samples T-test, which revealed no statistically significant differences (*p* = 0.224), while the gender distribution was also comparable (Pearson’s chi-squared, *p* = 0.798). Smoking differed significantly between the two groups (Pearson’s chi-squared, *p* = 0.011), with an approximately two-fold increased prevalence in OSCC cases. The patient and control groups were additionally compared with respect to smoking status categories using Pearson’s chi-squared test, which revealed a significant difference (*p* = 0.034), mainly driven by the moderate and heavy smoking categories, which were seven-fold and two-fold more prevalent in the OSCC population, respectively, compared to the controls. Hence, smoking was not only found to be different between cases and controls but also exhibited a dose-dependent pattern, suggesting its potential role as a confounding factor. While the overall alcohol consumption prevalence did not differ significantly between patients and controls (chi-squared test, *p* = 0.799), the comparison of the two groups regarding weekly drinking frequencies revealed a significant dose-dependent distribution, similarly to smoking (Pearson’s chi-squared, *p* = 0.005), placing alcohol consumption also under consideration as a potential confounder.

Table 4 summarizes the clinicopathological characteristics of the OSCC cohort, including the anatomical sites of the primary OSCC lesions and the tumor grade, categorized as well (G1), moderately (G2) and poorly differentiated (G3). Finally, patients were classified according to the clinical stage (I to IV) based on the TNM staging system. In agreement with established OSCC epidemiological data, moderate differentiation constituted the predominant histopathological grade (64.5%), whereas early-stage (I/II) and late-stage (III/IV) disease were almost equally represented (chi-squared goodness-of-fit test, *p* = 0.590). Regarding the anatomic position of the OSCC lesion, the majority (80.6%) were located within the oral cavity and were evenly distributed across intraoral anatomical subsites (chi-squared goodness-of-fit test, *p* = 0.888), while 19.4% corresponded to lip OSCC tumors.

### 2.3. Expression Profiling of Identified miRNAs 

#### 2.3.1. Expression Profiling of Identified miRNAs in Saliva of Patients and Controls

**Figure 10:**
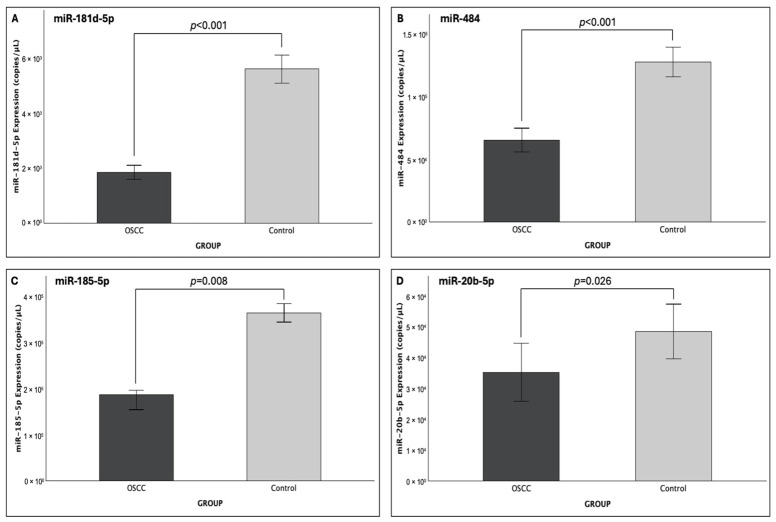
Comparative mean expression (copies/μL) of the studied miRNAs, which exhibited significant downregulation in OSCC patients compared to healthy controls. (**A**) Salivary miR-181d-5p demonstrated the greatest reduction of 67.1% (*p* < 0.001), followed by (**B**) miR-484 displaying 48.9% downregulation in patients (*p* < 0.001) and (**C**) miR-185-5p exhibiting a corresponding 48.7% reduction (*p* = 0.008), whereas (**D**) miR-20b-5p displayed a decrease of 27.4% in the OSCC group (*p* = 0.026).

The expression profiling of all bioinformatically selected miRNAs in salivary samples from the OSCC patients and healthy controls revealed a consistent downregulation pattern in the patient cohort compared to the controls. Among the analyzed molecules, miR-181d-5p exhibited a substantial 67.1% reduction in the saliva of OSCC patients compared to controls (FC: 0.329; *p* < 0.001), followed by miR-484, demonstrating a marked 48.9% decrease in in expression (FC: 0.511; *p* < 0.001), and miR-185-5p, which exhibited a 48.7% decrease in OSCC patients (FC: 0.513; *p* = 0.008). Finally, the levels of salivary miR-20b-5p exhibited a significant yet comparatively modest reduction of 27.4% in the OSCC group (FC: 0.726; *p* = 0.026) (Figure 10).

All studied miRNAs demonstrated the anticipated expression pattern of downregulation in OSCC, which was accordingly opposed to the established upregulation of their target genes. However, the extent of downregulation was not equal across all miRNAs, and it was not proportional to their target coverage. More specifically, miR-181d-5p, which exhibited panel coverage of 66.7% both at the disease level and in the stage-specific analysis, exhibited the greatest reduction in OSCC patients compared to controls. miR-185-5p, which scored 66.7% in the disease-level analysis and 83.3% in the stage-specific analysis—thus providing an intermediate mean target score of 75%—showed an approximately 20% smaller reduction, similarly to miR-484, which was exclusively yielded by the stage-specific model with panel coverage of 83.3%. Notably, miR-20b-5p, which was the highest-scoring in both analyses (mean panel coverage of 96.7%), exhibited a further attenuated decrease corresponding to a 20% smaller reduction compared to miR-484 and miR-185-5p and a 40% less pronounced reduction compared to miR-181d-5p (Table 5).

#### 2.3.2. Expression Profiling of Identified miRNAs in Saliva of Early-Stage Patients and Controls

**Figure 11:**
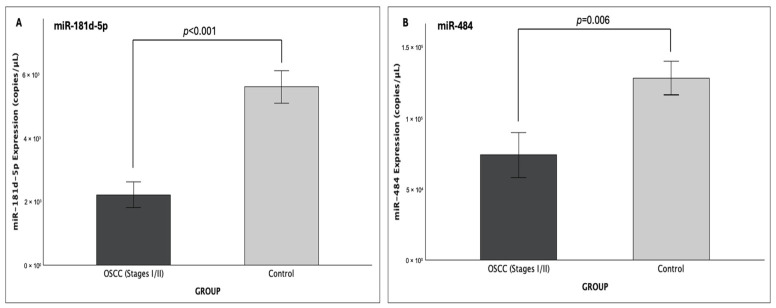
Comparative mean expression (copies/μL) of (**A**) miR-181d-5p and (**B**) miR-484, which exhibited significant downregulation in the subgroup of early-stage OSCC patients (stages I and II) compared to healthy controls (*p* < 0.01).

Given that the present study was designed to also identify miRNA molecules specific for early OSCC detection, the expression levels of the investigated miRNAs were secondarily compared between the subgroup of patients corresponding to OSCC early stages I and II (17 individuals) and the control group (31 individuals). Due to the early-stage subgroup’s reduced size, this analysis demonstrated 90% statistical power to detect very large effects, compared to the 97.2% power of the overall study design. The results indicated that all miRNAs followed the same downregulation trend in the OSCC subgroup, in line with the main findings of the study. However, only miR-181d-5p (independent-samples T-test; *p* < 0.001) and miR-484 (Mann–Whitney U test; *p* = 0.006) demonstrated statistically significant downregulation in the early-stage patient cohort compared to the controls (Figure 11). On the other hand, miR-20b-5p (Mann–Whitney U test; *p* = 0.077) and miR-185-5p (Mann–Whitney U test; *p* = 0.240) did not reach statistical significance, despite showing differential expression patterns. Hence, although it is possible that the other two miRNAs (miR-20b-5p and miR-185-5p) could achieve significant downregulation if using larger early-stage OSCC cohorts, the robust statistical significance achieved by miR-181d-5p and miR-484 supports their safe characterization as reliable salivary biomarkers for early-stage OSCC detection, even within such a small patient subgroup, when compared to healthy individuals. Moreover, it is interesting that these two miRNAs exhibited the highest diagnostic performance in the individually performed ROC analyses, while miR-484 was explicitly yielded as important through the stage-specific model. Hence, their presence in the combinatory panel, together with the rest of the strong experimental and bioinformatic evidence, further validates its robustness for early OSCC detection.

### 2.4. miRNA Expression Profiling in Relation to Lifestyle Factors

In order to investigate the potential confounding effect of smoking on miRNA expression, all participants (patients and controls) were categorized as smokers or non-smokers. The Mann–Whitney U test performed for each miRNA revealed no significant differences between the two groups (*p* > 0.05), thus indicating that smoking did not exert a confounding effect on the miRNA expression levels (miR-181d-5p: *p* = 0.353; miR-484: *p* = 0.379; miR-185-5p: *p* = 0.652; miR-20b-5p: *p* = 0.163). A corresponding analysis was conducted for alcohol intake, which similarly revealed no significant association between the alcohol consumption of the participants and miRNA expression, underlining the absence of a confounding influence (miR-181d-5p: *p* = 0.229; miR-484: *p* = 0.379; miR-185-5p: *p* = 0.331; miR-20b-5p: *p* = 0.966).

The majority of heavier smokers and alcohol users were included in the cohort of OSCC patients. Therefore, further analyses were employed in patients who were stratified by smoking and alcohol use, as well as by consumption status (light, moderate, and heavy). According to the applied parametric and non-parametric statistical tests, the expression levels of the studied salivary miRNAs remained unaffected by smoking (miR-181d-5p: *p* = 0.591; miR-484: *p* = 0.412; miR-185-5p: *p* = 0.741; miR-20b-5p: *p* = 0.312) or alcohol consumption (miR-181d-5p: *p* = 0.363; miR-484: *p* = 0.089; miR-185-5p: *p* = 0.097; miR-20b-5p: *p* = 0.244) or by the interaction of smoking and alcohol use, which was evaluated using a two-way general linear model (GLM) (miR-181d-5p: *p* = 0.430; miR-484: *p* = 0.140; miR-185-5p: *p* = 0.750; miR-20b-5p: *p* = 0.451). Regarding the possible effects of smoking and drinking habits on miRNA expression, the smoking frequency did not exert any dose-dependent influence on the levels of the studied miRNAs (miR-181d-5p: *p* = 0.935; miR-484: *p* = 0.569; miR-185-5p: *p* = 0.976; miR-20b-5p: *p* = 0.132), according to the performed one-way ANOVA and Kruskal–Wallis tests. In turn, despite the considerable proportion of heavy alcohol consumers within the patient cohort, no significant influence on miRNA levels was detected (miR-181d-5p: *p* = 0.728; miR-484: *p* = 0.743; miR-185-5p: *p* = 0.749; miR-20b-5p: *p* = 0.257), as indicated by the one-way ANOVA and Kruskal–Wallis tests.

### 2.5. miRNA Expression Profiling in Relation to Clinicopathological Parameters

Given the broad and representative distribution of tumor sites, OSCC differentiation grades and clinical stages within the patient cohort, their potential influence on miRNA expression was assessed. OSCC anatomical sites were evaluated at two levels, both as intraoral versus lip lesions and across the exact maxillofacial subsites. As indicated by the results of the Mann–Whitney U tests, the binary comparison showed no significant differences in miRNA expression (miR-181d-5p: *p* = 0.764; miR-484: *p* = 0.453; miR-185-5p: *p* = 0.980; miR-20b-5p: *p* = 0.250). Similarly, the multilevel analysis, performed using the one-way ANOVA and Kruskal–Wallis tests, did not identify any significant association between the tumor site and miRNA expression (miR-181d-5p: *p* = 0.856; miR-484: *p* = 0.805; miR-185-5p: *p* = 0.808; miR-20b-5p: *p* = 0.812).

The possible influence of the tumor grade on miRNA expression was evaluated by conducting a Kruskal–Wallis test, which did not reveal any significant differences in miRNA levels across the G1, G2 and G3 differentiation grades (miR-181d-5p: *p* = 0.105; miR-484: *p* = 0.245; miR-185-5p: *p* = 0.099; miR-20b-5p: *p* = 0.732). Similarly to the anatomical location, the clinical stage was approached at two levels. The first analysis encompassed a binary classification of early (I/II) versus late stage (III/IV) disease, which did not reveal any significant influence of the stage on miRNA levels (miR-181d-5p: *p* = 0.122; miR-484: *p* = 0.981; miR-185-5p: *p* = 0.416; miR-20b-5p: *p* = 0.766), according to the Mann–Whitney U and independent-samples T-tests. At the second level, miRNA expression was evaluated across the four distinct stages using the one-way ANOVA and Kruskal–Wallis tests, but, similarly, this did not reveal any stage-related patterns (miR-181d-5p: *p* = 0.220; miR-484: *p* = 0.424; miR-185-5p: *p* = 0.150; miR-20b-5p: *p* = 0.526).

### 2.6. Diagnostic Performance of Individual Salivary miRNAs and Combinatorial miRNA Panel

The identified salivary miRNA expression patterns were evaluated in terms of their diagnostic accuracy for the discrimination of OSCC patients and healthy individuals using receiver operating characteristic (ROC) curve analysis. The discriminative capacity of each individual miRNA, as well as that of the combinatory four-miRNA salivary panel (Figure 11), was quantified via the area under the curve (AUC), while the sensitivity and specificity levels were determined using the maximum Youden’s Index. Among the individually assessed miRNAs, miR-181d-5p demonstrated the highest (88.8%) diagnostic accuracy for saliva-based OSCC detection (AUC: 0.888; CI: 0.807–0.969; *p* < 0.001), while exhibiting excellent (90.3%) sensitivity (true positive rate) and 77.4% specificity (true negative rate). The second-best diagnostic performance (78.35%) was demonstrated by miR-484 (AUC: 0.783; CI: 0.668–0.899; *p* < 0.001), which achieved a sensitivity level of 77.4% and specificity of 74.2%. In third place, miR-185-5p exhibited diagnostic performance of 69.7% (AUC: 0.697; CI: 0.561–0.833; *p* = 0.004), with modest sensitivity of 48.4% but excellent specificity of 93.5%. Finally, miR-20b achieved the lowest yet significant diagnostic accuracy of 66.4% (AUC: 0.664; CI: 0.528–0.801; *p* = 0.018), accompanied by modest sensitivity of 51.6% and strong specificity of 83.9%.

**Figure 12:**
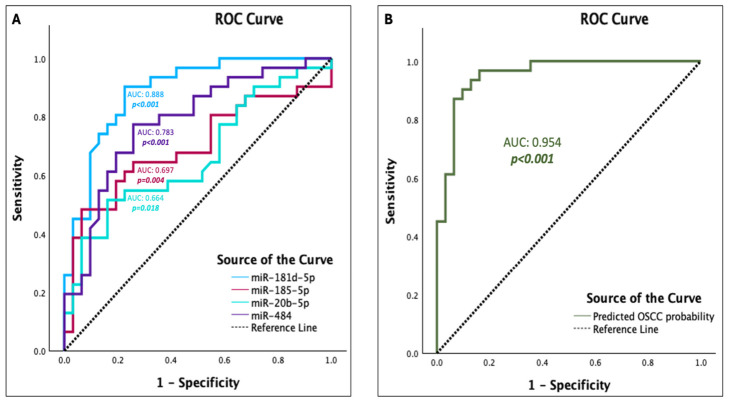
ROC curves depicting the diagnostic performance of (**A**) the individually studied miRNAs and (**B**) the developed four-miRNA combinatory panel for the saliva-based detection of OSCC regardless of lifestyle risk factors (smoking/alcohol), tumor site, grade (G1–G3) and disease stage (I-IV). The dashed diagonal line in both graphs indicates the reference line (no discrimination/AUC: 0.5), while the different curves and corresponding AUC and *p*-values are illustrated by distinct colors, as indicated in the figure legends.

A binary logistic regression model was constructed to combine the identified miRNAs into a saliva-based diagnostic panel. The derived predicted probabilities were subsequently subjected to ROC curve analysis, as well as sensitivity/specificity assessment, in order to evaluate the panel’s diagnostic performance. The developed four-miRNA panel demonstrated excellent saliva-based discriminatory performance (95.4%) between OSCC and normal mucosae (AUC: 0.954; CI: 0.905–1.003; *p* < 0.001) (Figure 12). At the optimal cutoff, as determined by Youden’s Index, the panel achieved excellent detection sensitivity of 96.8%, while maintaining high specificity of 83.9%. The bootstrap analysis of the developed miRNA panel (1000 resamples) yielded a mean AUC of 0.9542, almost identical to the original estimate, and a slightly narrower confidence interval (CI: 0.894–0.995), therefore confirming the robustness and stability of the panel and indicating that the observed diagnostic performance is not driven by specific sample configurations.

**Figure 13:**
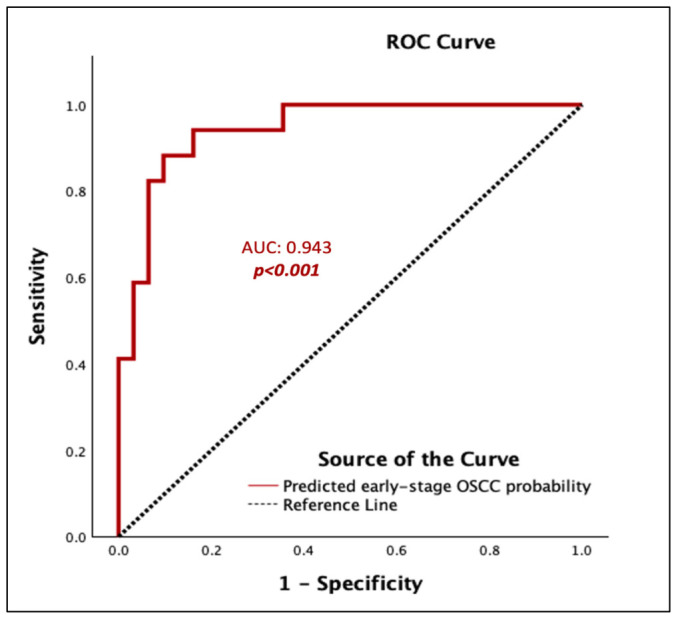
ROC curve depicting the diagnostic performance of the developed four-miRNA combinatory panel for the saliva-based detection of OSCC in the early stages I and II. The dashed diagonal line indicates the reference line (no discrimination/AUC: 0.5), while the curves and the corresponding AUC are illustrated in dark red, as indicated in the figure legend.

Finally, given the study’s design, intending to also reveal miRNA biomarkers for early OSCC detection, a secondary ROC analysis was performed to evaluate the diagnostic accuracy of the developed panel, including the predicted probabilities corresponding to early-stage OSCC patients (stages I and II) and controls. The analysis yielded strong diagnostic performance (94.3%) for early OSCC detection (AUC: 0.943; CI: 0.881–1.005; *p* < 0.001) (Figure 13). At the optimal cutoff, as defined by Youden’s Index, the panel demonstrated 88.2% sensitivity for the detection of early-stage OSCC lesions, as well as excellent specificity of 97% for stages I and II. Bootstrap validation in the early-stage subgroup (1000 resamples) indicated a mean AUC of 0.941 (CI: 0.871–0.9924), supporting the stability of the panel for early detection, despite the reduced sample size of the OSCC subgroup.

## 3. Discussion

OSCC represents the sixth most prevalent malignancy worldwide, with consistently increasing incident rates [1,3,4]. It is characterized by profound aggressiveness, associated with high rates of lymph node involvement, metastasis and recurrence [10,11]. Despite significant advances in treatment and histopathological assessment, it remains associated with persistently high 5-year mortality rates that reach up to 60% [17,18,19]. This is largely attributable to extensive diagnostic delays, mainly stemming from the asymptomatic early histological stages, as well as the significantly time-consuming diagnostic workflows [9,16]. As a result, more than half of patients present with already advanced disease (stages III and IV), which is associated with a significantly poor prognosis, in contrast to early-stage diagnosis (stages I and II), which confers a markedly improved likelihood of survival (up to 80%) [5,9,16,17,24,25].

It follows that the establishment of reliable biomarkers for the routine assessment of the oral mucosa is imperative. Given its direct contact with OSCC tumor sites, saliva represents the most reliable and accessible biofluid for non-invasive oral epithelial monitoring. This approach requires the identification of disease-specific, reliable biomarkers, which, however, have not yet been established [9,17,29,31]. miRNAs, which are key epigenetic regulators of gene expression, represent ideal candidates given their stability in biofluids and their characteristic disease-reflecting expression patterns across different pathologies [9,31,33]. Building upon the well-established genetic landscape of OSCC pathogenesis [18,35,36], our group has developed two integrative genetic/epigenetic bioinformatic models for the identification of the most specific miRNAs for OSCC, both at a disease and a stage-specific level [18,25].

**Figure 14:**
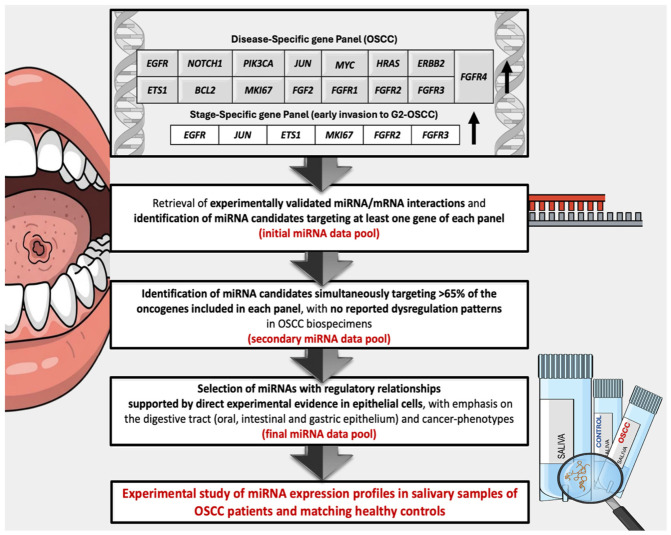
Flowchart summarizing the bioinformatic pipeline pursued for the selection of OSCC-specific miRNA molecules for an experimental study on salivary samples of patients with OSCC and matching healthy controls. Upward arrows indicate the upregulation of the genes included in each panel during OSCC progression, both in a disease- and a stage-specific context.

Here, by employing our two bioinformatic models in a novel combinatorial strategy (Figure 14), we aimed to identify OSCC-specific miRNAs with diagnostic potential for the reliable saliva-based detection of OSCC, from the earliest presymptomatic stage of early invasion. More specifically, we developed two customized gene panels—one incorporating the 15 primarily upregulated oncogenes in OSCC [18] and one encompassing the six regulated genes that genetically define the malignant stages of sequential oral carcinogenesis (early invasion, well differentiated and moderately differentiated OSCC) [36,37]—to provide a complementary level of resolution.

**Figure 15:**
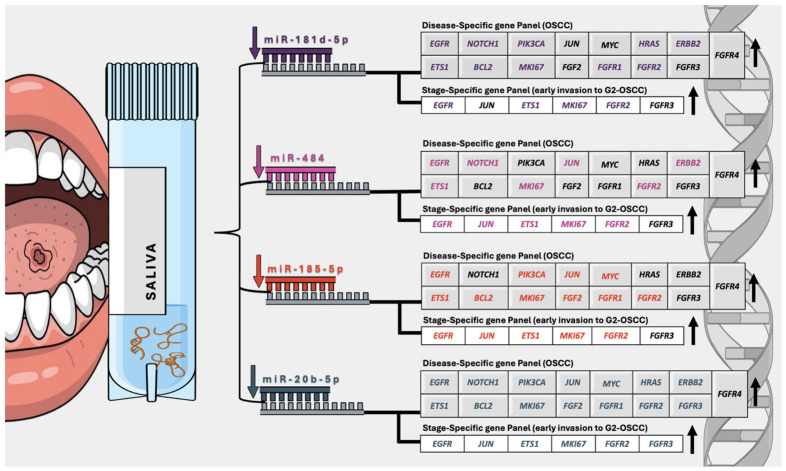
Integrative graphical summary of the observed dysregulation patterns of each identified OSCC-specific miRNA in the saliva of patients compared to that of healthy individuals. Genes whose expression is regulated by each miRNA are depicted using a corresponding color across both analytical panels generated for the disease and in a stage-specific bioinformatic analyses. These comprise the most significant genes with established overexpression in OSCC at a disease level, as well as those whose upregulation defines the molecular signature of the sequential malignant stages of oral carcinogenesis, spanning from early invasion to moderately differentiated OSCC tumors. Expression patterns are indicated by directional arrows, with upward arrows indicating upregulation and downward arrows denoting downregulation. Black arrows indicate the expression pattern of the genes included in each panel, while colored arrows correspond to the respective miRNAs and match the colors used for their representation in the figure.

Through a multistep analytical workflow, we managed to identify four OSCC-specific miRNAs (miR-20b-5p, miR-484, miR-185-5p and miR-181d-5p), which were experimentally shown to simultaneously regulate over 65% of the genes in each panel, through direct experiments, primarily in epithelial cells, with a focus on the digestive tract (oral, intestinal and gastric epithelium) and/or in cancer phenotype models, while retaining no previously reported expression patterns in OSCC-related biospecimens to date (Figure 15). The overlap of three miRNAs (miR-20b-5p, miR-185-5p and miR-181d-5p) identified across both analytical frameworks, together with miR-484, which was yielded in a stage-specific manner from the stage of early invasion to moderately differentiated tumors, indicated a robust set of candidates for saliva-based investigation, which is particularly promising in terms of capturing and reflecting OSCC beginning at its presymptomatic initiation.

The selected miRNAs were studied in a clinically representative cohort of patients in comparison to matched healthy controls. As hypothesized, the selected miRNAs demonstrated significant salivary downregulation (up to 67.1%) in OSCC patients compared to controls, which was consistent with the expected inverse regulatory relationship with their upregulated gene targets (miR-181d-5p, *p* < 0.001; miR-484, *p* < 0.001; miR-185-5p, *p* = 0.008; and miR-20b-5p, *p* = 0.026).

Although all studied miRNAs were significantly downregulated in the saliva of OSCC patients, their magnitudes of reduction were not proportional to their target scores. The most profound downregulation in OSCC saliva (67.1%, *p* < 0.001) was exhibited by miR-181d-5p, which exhibited the lowest target coverage in both analyses (66.7%), followed by miR-484, which scored 83.3% in the stage-specific analysis and exhibited a nearly 50% reduction (*p* < 0.001), as well miR-185-5p with a mean target score of 75% and similar downregulation of around 50% (*p* = 0.008). In contrast, miR-20b-5p, with an overall near-perfect mean target score (96.7%), demonstrated a disproportional yet significant degree of expressional suppression (27.4%, *p* = 0.026).

According the ROC-based evaluation, individual miRNAs demonstrated variable diagnostic accuracy (66.4–88.8%) and differing sensitivity (48.4–90.3%) and specificity (74.2–93.5%) levels. However, their combined evaluation as a saliva-based diagnostic panel yielded outstanding discriminative performance of 95.4% (AUC: 0.954%, *p* < 0.001) between OSCC and normal mucosae, as well as excellent sensitivity (96.8%) and specificity of 83.9%%, thus underscoring the complementary biological and diagnostic value of each incorporated miRNA.

More specifically, certain miRNAs, such as miR-181d-5p and miR-484, contributed predominantly to the sensitivity, whereas miR-20b-5p and miR-185-5p provided high levels of specificity, collectively enhancing the overall performance of the panel. In addition, in light of the fact that the potential lifestyle and clinicopathological factors did not demonstrate any confounding influence on miRNA expression, the developed four-miRNA panel exhibits robust and stable diagnostic performance, independently of smoking, alcohol intake, the tumor site, the differentiation grade (G1–G3) and the clinical disease stage (I–IV), thereby encompassing the complete clinicopathological spectrum of oral oncogenesis.

Finally, miR-181d-5p (*p* < 0.001) and miR-484 (*p* = 0.006) were observed to be significantly downregulated in early-stage patients (I/II) compared to controls, thereby underscoring their robustness as reliable salivary biomarkers for OSCC detection even in the earliest stages. It is worth mentioning that these two miRNAs exhibited the highest diagnostic performance in the individually performed ROC analyses, while miR-484 was explicitly yielded as important through the stage-specific analysis. Due to the reduced size of the subcohort, the lack of statistical significance for the other two molecules, especially miR-20b-5p (*p* = 0.077), might be attributable to the reduced power to detect less profound differences, rather than the absence of an actual effect. Hence, the presence of miR-181d-5p and miR-484, which clearly dominated the diagnostic signal in the combinatory miRNA-panel, together with the rest of the strong experimental and bioinformatic evidence, further supports its clinical relevance for reliable OSCC detection, even in the earliest stages. The latter was confirmed by the results of the corresponding ROC-based evaluation of the panel’s performance in the context of early-stage OSCC, where it demonstrated 94.3% accuracy for the saliva-based detection of stage I and stage II lesions, with sensitivity of approximately 88% and excellent specificity of 97%.

The consistent performance of the panel following bootstrap internal validation in both overall OSCC detection of any stage and grade and in the early-stage cohort strongly supports the robustness of the findings and underlines that the observed diagnostic accuracy is unlikely to be driven by random variation, thereby excluding the risk of significant overfitting.

A shared target of all studied miRNAs is *MKI67*, a well-established proliferation marker that is significantly overexpressed in OSCC and indicative of its existence from the earliest stages [38], including the malignant transformation of precancerous lesions [39]. This observation further supports the capacity of the combinatorial miRNA panel to reflect oral carcinogenesis. Three out of four miRNAs (miR-181d-5p, miR-185-5p and miR-20b-5p) also regulate the expression of *BCL2*, a key anti-apoptotic marker that is overexpressed in OSCC tumors and is associated with the malignant transformation of oral dysplasia, with highly promising utility in histopathological assessment [40,41].

The expression of both markers is not only upregulated but also proportionally increases with OSCC progression, thereby reflecting the status of oncogenesis [41]. Their shared regulation by the identified miRNAs, in combination with their significant OSCC downregulation and high diagnostic performance, further strengthens their diagnostic reliability. Hence, the developed panel could represent a promising alternative to marker-based histopathological diagnosis, enabling non-invasive OSCC detection but also indirectly leveraging the dysregulation of established histopathological markers that indicate the two main axes of carcinogenesis (namely proliferation and anti-apoptosis).

As previously mentioned, the observed downregulation of the examined miRNAs was a priori conceptually supported by the well-documented upregulation of their several oncogenic gene targets in OSCC progression. The simultaneous rise in the levels of multiple transcripts within the tumor microenvironment is highly likely to contribute to the functional exhaustion and therefore significant diminishing of their miRNA regulators, which are forced to counterbalance a multitude of upregulated targets at once. This possibly accounts for the observed reduction in their salivary expression, while creating a feedback loop of inadequate epigenetic suppression that might further contribute to the malignant progression of the OSCC tumor.

The apparent paradox stemming from the disproportionate modest downregulation observed for miR-20b-5p relative to its target burden might be indicative of a possibly dual role of this particular miRNA. While its significant downregulation in the OSCC cohort is consistent with its disease-specific targets and their concurrent upregulation, its function as a broad-spectrum regulatory molecule within multiple signaling networks might impose selective constraints that prevent its further diminishing. This notion is highly consistent with the documented function of miR-20b-5p as an epigenetic inhibitor of inflammation and apoptosis in the digestive tract, with reported involvement in autophagy-mediated angiogenic pathways [42]. Thereby, while its disease-specific downregulation might be indicative of OSCC presence or even contribute as a pathogenetic mechanism of tumor progression, it may also exert a reactive (yet inadequate) tumor-suppressing effect.

This is further reflected by the context-dependent role of miR-20b-5p across different cancer types, with contradicting roles as an oncomiR or a tumor suppressor miRNA [43], likely highlighting disease-specific differences in its target landscape. The present bioinformatic and experimental study indicates a tumor-suppressive role of miR-20b-5p in OSCC. This notion is supported by its documentation as a tumor suppressor miRNA in colon cancer, which exhibits significant genetic resemblance to OSCC and other cancers of the digestive tract [44,45].

Regarding the remaining three studied molecules showing substantial downregulation (48.7–67.1%; *p* < 0.01) in the saliva of OSCC patients, they mainly scored higher in the stage-specific analysis, which solely encompassed core genetic drivers of oral malignant transformation, thereby highlighting a possibly pathogenetic and OSCC-reflecting rather than reactive role. Their specificity for the disease is further supported primarily by miR-484, which was only yielded by the stage-specific analysis and ranked second in diagnostic performance (78.3%, *p* < 0.001).

Specifically, regarding miR-484, while exhibiting an inconsistent pattern of expression across different pathologies and significant upregulation in a variety of malignancies, such as brain and cervical carcinomas [46], it demonstrates a marked downregulation pattern solely in gastric cancer [47]. The latter leads to the upregulation of the PI3K/AKT oncogenic pathway and is associated with significantly poorer prognostic outcomes, additionally corroborating its tumor-suppressive function in the digestive tract [47,48], as suggested in the present study. This notion is in line with its role in colorectal cancer as a tumor suppressor, where its reduced expression has been associated with tumor progression and increased metastatic potential [49].

To our knowledge, the present study is the first to highlight the overall involvement and diagnostic relevance of miR-181d-5p in OSCC, demonstrating profound downregulation (67.1%, *p* < 0.001) in patient saliva and the highest diagnostic performance (88.8%, *p* < 0.001) among the studied molecules. While its role in human cancers remains unclear [50], recent strong evidence from the genetically similar context of colorectal cancer supports a tumor-suppressive and therapeutically relevant function of miR-181d-5p [51], thereby providing further support for its OSCC-specific role, as highlighted in the present study.

Finally, while the role of miR-185-5p in OSCC has not been elucidated, functional studies underline its ability to restore cisplatin sensitivity in OSCC and colorectal carcinomas [52]. Its reduced levels have been associated with significantly enhanced tumor growth and metastatic potential in neoplasms of the digestive tract, including colorectal and pancreatic carcinomas [53,54,55]. A tumor-suppressive role has also been shown in breast cancer, through the induction of G1/S phase arrest [56], as well as in the contexts of myeloid leukemia and prostate cancer, where its induced upregulation inhibited malignant progression by promoting apoptosis [57,58]. Finally, a very recent study in precancerous oral leukoplakia reported the significant downregulation of miR-185 in salivary exosomes, demonstrating its association with malignant transformation to OSCC [59]. This independent finding further corroborates our overall results and provides additional support for the early stage-specific framework of the present study.

### Limitations and Future Perspectives

Despite the fact that the present study demonstrated high statistical power of 97.2% to detect large effects (Cohen’s *d* = 1) and the subject groups were equally distributed, the relatively modest size of the studied sample, encompassing 62 individuals, remains a limitation. In addition, despite the robustness indicated by bootstrap internal validation, a residual possibility of overfitting cannot be entirely excluded due to the limited size of the studied cohorts, particularly in the early-stage subgroup analysis. Moreover, while the early-stage findings are supported by both strong statistical evidence and extensive bioinformatic rationale, the reduced number of early-stage patients limited the detection of more subtle effects for individual miRNAs, such as miR-20b-5p. Therefore, further validation in larger independent populations is required to externally validate the robustness of the developed panel, as well as the exact cutoffs of miRNA expression for diagnostic utilization. In addition, although no significant association with potential confounders was detected, larger patient subgroups corresponding to each differentiation grade and clinical stage would be useful for the enhanced validation of the panel’s stability across histopathological subgroups. This would be particularly important for early-stage cases and well-differentiated tumors, which retain the closest resemblance to the normal epithelium.

## 4. Materials and Methods

### 4.1. Bioinformatic Identification of Novel OSCC-Specific miRNAs for Experimental Study

In the present study, a combination of our disease- and stage-specific bioinformatic models [18,25] was applied to identify OSCC miRNAs that are predicted to remain dysregulated across the full spectrum of oral oncogenesis, from the presymptomatic stage of early invasion, and to finally select the most promising candidates for experimental evaluation. Given that the majority (around 75%) of genes critical for OSCC pathogenesis are oncogenes [18], and that the malignant stages of oral oncogenesis are primarily driven by oncogene overexpression [25,36,37], we designed both disease- and stage-specific bioinformatic analyses based on custom oncogene panels. The customized panels covered both the disease as a whole and the dysregulated genetic signature of each malignant histological stage, according to our group’s animal system of sequential oral carcinogenesis [36,37]. Candidate miRNAs with potentially high OSCC specificity were identified based on (a) their experimentally validated regulatory interactions with the majority of oncogenes in each panel and (b) the absence of previously documented expression patterns in OSCC-derived specimens in order to ensure novel findings.

Based on this approach, miRNAs that concurrently regulate multiple oncogenic drivers were expected to exhibit significant downregulation in OSCC patients, in accordance with the well-established inverse relationship between miRNA and gene-target expression. The retrieval of experimentally validated miRNA/mRNA interactions was combinatorially performed via the utilization of miRNet 2.0 (Xia Lab, McGill University, Montreal, QC, Canada), DIANA-TarBase.v.9 (DIANA Lab, University of Thessaly, Volos, Greece), incorporating DIANA-microT-CDS, as well as miRTarBase (The Chinese University of Hong Kong, Shenzhen, China).

At the disease level, a 15-gene panel was created, encompassing all predominant oncogenes that have been established as key contributors to OSCC progression through their overexpression (*EGFR*, *NOTCH1*, *PIK3CA*, *JUN*, *MYC*, *HRAS*, *ERBB2*, *ETS1*, *BCL2*, *MKI67*, *FGF2*, *FGFR1*, *FGFR2*, *FGFR3* and *FGFR4*), based on the relevant literature [18]. In order to identify miRNAs that possibly covered the whole spectrum of oral carcinogenesis, we incorporated genomic data from our group’s acknowledged experimental model, which encompasses every distinct stage, from hyperkeratosis, hyperplasia, dysplasia and early invasion to well and moderately differentiated OSCC, and presents the dysregulated genetic signature of each OSCC stage [37]. Among the consecutive stages of oral oncogenesis, only those occurring after the point of malignant transformation were selected. Genes reported to be upregulated during each malignant stage were annotated, resulting in the development of a 6-oncogene panel (*EGFR*, *JUN*, *ETS1*, *MKI67*, *FGFR2*, *FGFR3*), representing OSCC progression from the presymptomatic stage of early invasion through fully developed tumors of different grading.

By employing the aforementioned panels as genomic input, two miRNA/target interaction analyses were performed using miRNet 2.0, resulting in the construction of two distinct miRNA regulatory networks, comprising all available experimentally validated interactions between miRNAs and each gene of the respective panel. Among the multitude of miRNAs represented in each regulatory network, those simultaneously targeting >65% of the oncogenes included in each panel were selected and subsequently screened in the PubMed database to determine whether their expression has been previously investigated in OSCC-derived specimens (accessed on 10 November 2025). miRNAs with no reported expression data, or with evidence limited to functional studies suggesting a tumor-suppressing role, were ultimately selected. Their interactions with the OSCC-related oncogenes were further investigated utilizing the DIANA-TarBase and miRTarBase tools. To ensure high-confidence and relevant miRNA/mRNA complexes, inclusion criteria were applied. Following restriction to experimentally validated interactions, a secondary filter was implemented, requiring at least 50% of each yielded miRNA’s regulatory relationships to be supported by direct experimental evidence in epithelial cells, with an emphasis on the digestive tract (oral, intestinal and gastric epithelium) and cancer phenotype models. The miRNA molecules fulfilling all filtering requirements were selected for experimental assessment in the saliva of OSCC patients and matching healthy controls.

Despite partial overlap between the developed panels, both analyses were intentionally employed to provide complementary levels of resolution. The disease-level strategy enabled the identification of miRNAs regulating the broader landscape of oncogenes involved in OSCC, whereas the stage-specific approach offered refined insight into the exact mechanisms of tumor progression that characterize the malignant stages of oral oncogenesis. Hence, the present hierarchical framework aimed at enhancing biological specificity and capturing miRNAs whose downregulation in saliva can be expected from the presymptomatic stage of the early invasion of OSCC cells.

### 4.2. Sample Collection

The study protocol was approved by the Bioethics Committee of the Department of Oral and Maxillofacial Surgery (1-22-11-2021) and the Scientific Council (A7-08-02-2022) of Attikon University General Hospital. Salivary samples were collected from patients with OSCC and from healthy individuals in the University Department of Oral and Maxillofacial Surgery at Attikon Hospital. Inclusion criteria for patients included a clinical and histological diagnosis of OSCC lesions of the oral cavity and lips that were active during sample collection, as well as the absence of any concurrent oral lesions of different etiology or any prior OSCC-related treatment. The control population included healthy individuals of comparable age and gender, with clinically normal oral mucosae, no history of OSCC or oral potentially malignant disorders (OPMDs) and no recent infections or systemic inflammatory diseases, aiming to minimize potential confounding effects on salivary miRNA expression.

Basic demographic characteristics, including age and gender, were collected from all participants, along with a complete medical history and documentation of lifestyle factors that are strongly associated with OSCC pathogenesis, such as smoking and alcohol consumption. The latter were recorded both as binary variables (yes/no) and as frequency measures, expressed as cigarette packs and alcoholic beverages per week, respectively. From the group of patients, clinicopathological data were recorded, including the anatomical site (oral cavity or lip) and exact intraoral position of the OSCC lesion, as well as the tumor differentiation grade (G1–G3) and OSCC stage (I–IV). Informed written consent was obtained from all enrolled participants for saliva sampling and subsequent molecular analysis and the collection of personal information and clinicopathological data, as well as for the photographic documentation of the OSCC lesions in the patient group.

From each participant, 2.0 mL of whole, unstimulated saliva was collected using saliva RNA collection and preservation devices (Norgen Biotek, Thorold, ON, Canada), followed by the addition of the respective aqueous buffer, according to the manufacturer’s instructions, to facilitate cellular lysis and RNA preservation. Prior to saliva collection, all participants refrained from food and water intake, as well as from performing oral hygiene procedures, for at least one hour and rinsed their mouths with water 15 min before sampling. Saliva samples were homogenized immediately after collection by manual agitation and were optically observed to ensure the absence of blood contamination. The preserved samples were stored at 4 °C until downstream processing.

### 4.3. Salivary RNA Isolation and miRNA Reverse Transcription

A volume of 250 μL from each collected salivary sample was used for total RNA extraction upon the addition of 150 μL of sterile 1× PBS of pH = 7.0–7.5 (Capricorn Scientific, Ebsdorfergrund, Germany). RNA isolation was performed using the Saliva/Swab RNA Purification Kit (Norgen Biotek, Thorold, ON, Canada), according to the manufacturer’s instructions for preserved saliva specimens. During the lysis step of the protocol, a synthetic exogenous miRNA molecule (cel-miR-39-3p) was included as a spike-in control to monitor the consistency of RNA extraction and downstream analyses (Qiagen, Hilden, Germany). Finally, to increase the obtained miRNA concentration, RNA was eluted from the silica membrane in 25 μL, instead of the recommended 50 μL elution volume.

Directly following RNA extraction, samples were assessed regarding the concentration in nanograms per microliter (ng/μL) and purity using a Quawell Q6000 UV-Vis Spectrophotometer (Quawell, San Jose, CA, USA). RNA input concentrations were normalized across all studied samples to ensure technical consistency prior to miRNA polyadenylation and reverse transcription (RT), carried out in a single-step reaction using the miRCURY LNA RT Kit (Qiagen, Hilden, Germany). The 10 μL polyadenylation/RT reaction involved the addition of 2 μL 5× RT-SYBR-Green Reaction Buffer, 2 μL RNA template, 1 μL 10× RT-Enzyme-Mix and 5 μL of nuclease-free water and was performed on a MyCycler thermal cycler (Bio-Rad Laboratories Inc., San Francisco Bay Area, CA, USA). The two-step thermocycling protocol involved 60 min incubation at 40 °C, followed by an inactivation step at 95 °C and a final hold at 4 °C. Following overnight storage at −20 °C, the cDNA samples were thawed and processed the following day.

### 4.4. Absolute Quantification of miRNA Expression by Quantitative Real-Time PCR

Quantification of the selected miRNAs (miR-20b-5p, miR-181d-5p, miR-185-5p and miR-484) was performed by real-time quantitative PCR (RT-qPCR) on a QuantStudio^TM^ 3 Real-Time PCR system (Thermo Fisher Scientific Inc., Waltham, MA, USA). The levels of the studied miRNAs were determined by absolute quantification using the standard curve method and expressed as the copy number per μL of each PCR reaction (copies/μL).

The construction of standard curves involved the custom design of synthetic RNA oligonucleotides (Eurofins Genomics, Ebersberg, Germany) corresponding to the mature sequences of the studied miRNA molecules as annotated in the miRBase.org miRNA database (hsa-miR-484: MIMAT0002174; hsa-miR-20b-5p: MIMAT0001413; hsa-miR-181d-5p: MIMAT0002821; hsa-miR-185-5p: MIMAT0000455). Standard curves were generated using 2-fold serial dilutions of the respective reverse-transcribed oligonucleotides, covering at least 11 concentration points for each miRNA, while demonstrating high linearity with coefficient of determination (R^2^) values of 0.9982–0.9992 (>0.99) and amplification efficiencies ranging from 97.16% to 99.12%.

RT-qPCR reactions were conducted using SYBR^®^ Green chemistry and ROX as a passive reference dye, with each well containing a 10 μL reaction mixture of 5 μL of miRCURY SYBR Green Master Mix, 0.05 μL of ROX dye, 3.95 μL of cDNA template and 1 μL of miRCURY LNA miRNA PCR Assay, suitable for the detection of each miRNA (Qiagen, Hilden, Germany). The qPCR thermal protocol was designed in “fast mode” and incorporated an initial PCR heat activation step for 2 min at 95 °C (4.14 °C/s), followed by 45 cycles of 95 °C for 10 s and 56 °C for 1 min and a final melting curve step spanning 60–95 °C. Samples of standard concentrations were used as a quality control measure to ensure technical consistency between runs, and no systematic batch effects were observed during the experimental process. Threshold cycles (Ct values) were obtained through the instrument’s built-in Design and Analysis software (v1.5.1), with all samples reaching amplification below the threshold of 40 cycles that was set for reliable detection, thus confirming adequate miRNA expression levels and assay reliability.

### 4.5. Statistical Analysis

Statistical analyses were performed using version 31.0.2.0 of SPSS Statistics (IBM Corp., Armonk, NY, USA). Normality was evaluated by the employment of the Shapiro–Wilk test for continuous values, based on the sample sizes (<50) for each cohort and studied subgroup. Comparisons between independent groups were conducted using the independent-samples T-test or Mann–Whitney U test, depending on the distribution of the values. Specifically, comparisons between more than two groups were performed using one-way ANOVA or the Kruskal–Wallis test, while the Jonckheere–Terpstra (JT) test was additionally applied in cases of ordered groups.

General linear models (GLMs) were employed for the evaluation of the combined effects of different factors on the miRNA expression levels. Comparative results are presented in bar charts with corresponding error bars indicating variability around the estimated mean values. An a priori power analysis was performed to estimate the statistical power of the present study design (31 patients and 31 controls), assuming a two-sided significance level of α = 0.05. The latter demonstrated 97.2% power of the study to detect very large effects (Cohen’s *d* = 1). The level of statistical significance was set at *p* < 0.05 (two-tailed).

The diagnostic accuracy of each individual miRNA and the multi-miRNA panel was assessed by employing receiver operating characteristic (ROC) curve analyses with OSCC as the positive state, while an additional subgroup analysis was performed for the evaluation of the panel’s diagnostic performance regarding early-stage disease (stages I and II). The latter included the calculation of the area under the curve (AUC) to evaluate the discriminative performance, while sensitivity and specificity were determined based on Youden’s Index. Internal validation was performed using bootstrapping in the R statistical computing environment (R Foundation for Statistical Computing, Vienna, Austria), through the employment the pROC package and the subsequent generation of 1000 resamples with replacement. For each iteration, the AUC was calculated based on the predicted probabilities of the panel for OSCC detection, yielding a mean estimate for each examined setting. Internal validation was performed both for the validation of the overall diagnostic performance of the miRNA panel in OSCC detection and for the saliva-based detection of early-stage disease.

## 5. Conclusions

The present research indicates the salivary downregulation of miR-181d-5p, miR-484, miR-185-5p and miR-20b, possibly representing a disease-specific epigenetic signature for OSCC. Through a two-tier bioinformatic analysis, these miRNAs were identified as specific for capturing the full spectrum of oral carcinogenesis as they simultaneously regulate the majority of the key genes overexpressed during OSCC progression, which genetically define its pathogenesis from the histopathological stage of early invasion. Their anticipated pattern of downregulation, in contrast to the overexpression of their target oncogenes, was validated in the saliva of OSCC patients compared to healthy controls. From a diagnostic angle, the combinatorial salivary four-miRNA panel, consisting of miR-181d-5p, miR-484, miR-185-5p and miR-20b, achieved excellent accuracy (95.4%; *p* < 0.001) for the discrimination of OSCC from the normal oral epithelium. Importantly, miRNA expression levels were not significantly influenced by potential lifestyle confounders, such as smoking or alcohol consumption, or by their interaction or the respective frequency of use.

Furthermore, besides their strategic bioinformatic selection to capture sequential oral oncogenesis from the earliest stages, the expression of all miRNAs remained unaffected by the tumor grade (G1–G3) and clinical disease stage (I–IV), suggesting the cancer-specific origins of their dysregulation. Collectively, these findings suggest that the proposed panel maintains consistent diagnostic performance for the saliva-based detection of OSCC irrespective of histopathological or lifestyle confounders. This highlights its potential as a robust and widely applicable diagnostic tool for the detection of OSCC in light of a suspicious lesion or during routine monitoring of the oral mucosa within the context of preventative screening. We anticipate that this experimental validation of our disease- and stage-specific bioinformatic models may constitute a meaningful addition to the ongoing global efforts to improve OSCC prognostic outcomes.

Beyond their diagnostic value, the findings of the present research may also provide insights into the underlying mechanisms of OSCC progression that encompass the interplay between genetics and epigenetics. More specifically, the observed significant downregulation of the selected miRNAs is highly consistent with the well-established inverse relationship between miRNA molecules and their gene targets and may also reflect their functional exhaustion in light of multiple overexpressing oncogenic targets. Furthermore, their substantial reduction in the OSCC cohort is in line with the recently elucidated tumor-suppressive roles of all four miRNAs in cancers of the digestive tract, which share genetic similarities to OSCC. Collectively, these observations, combined with their bioinformatically documented specificity to OSCC tumorigenesis, suggest that miR-181d-5p, miR-484, miR-185-5p and miR-20b-5p may not only serve as diagnostic biomarkers but also exhibit potential mechanistic relevance in the progression of oral oncogenesis.

## Figures and Tables

**Table 1:** Overview of the results of the disease-level bioinformatic analysis that led to the identification of miR-20b-5p, miR-185-5p and miR-181d-5p, which simultaneously regulated 66.7–93.3% of the oncogenes included in the developed disease-specific panel (*EGFR*, *NOTCH1*, *PIK3CA*, *JUN*, *MYC*, *HRAS*, *ERBB2*, *ETS1*, *BCL2*, *MKI67*, *FGF2*, *FGFR1*, *FGFR2*, *FGFR3* and *FGFR4*). Target scores represent the number of genes regulated within experimentally validated miRNA/mRNA interactions, primarily in epithelial cells, with a focus on the digestive tract and malignant phenotypes.

	Disease-Level Analysis Results	
Identified miRNA	OSCC-Upregulated Target Genes	Target Score (*n*/15, %)
miR-20b-5p	*EGFR*, *NOTCH1*, *PIK3CA*, *JUN*, *MYC*, *HRAS*, *ERBB2*, *ETS1*, *BCL2*, *MKI67*, *FGF2*, *FGFR1*, *FGFR2*, *FGFR3*	14/15 (93.3%)
miR-185-5p	*EGFR*, *PIK3CA*, *JUN*, *MYC*, *ETS1*, *BCL2*, *MKI67*, *FGF2*, *FGFR1*, *FGFR2*	10/15 (66.7%)
miR-181d-5p	*EGFR*, *NOTCH1*, *PIK3CA*, *HRAS*, *ERBB2*, *ETS1*, *BCL2*, *MKI67*, *FGFR1*, *FGFR2*	10/15 (66.7%)

**Table 2:** Overview of the results of the stage-specific bioinformatic analysis that led to the identification of miR-20b-5p, miR-484, miR-185-5p and miR-181d-5p, which simultaneously regulated 66.7–100.0% of the genes encompassed in the developed stage-specific panel (*EGFR*, *JUN*, *ETS1*, *MKI67*, *FGFR2* and *FGFR3*). Target scores represent the number of genes regulated within experimentally validated miRNA/mRNA interactions, primarily in epithelial cells, with a focus on the digestive tract and malignant phenotypes.

	Stage-Specific Analysis Results	
Identified miRNA	Stage-Specific Upregulated Target Genes (Early Invasion to Well-Differentiated OSCC)	Target Score (*n*/15, %)
miR-20b-5p	*EGFR*, *JUN*, *ETS1*, *MKI67*, *FGFR2*, *FGFR3*	6/6 (100.0%)
miR-484	*EGFR*, *JUN*, *ETS1*, *MKI67*, *FGFR2*	5/6 (83.3%)
miR-185-5p	*EGFR*, *JUN*, *ETS1*, *MKI67*, *FGFR2*	5/6 (83.3%)
miR-181d-5p	*EGFR*, *ETS1*, *MKI67*, *FGFR2*	4/6 (66.7%)

**Table 3:** Summary of demographic characteristics (age/gender) of the studied groups (OSCC patients and controls), as well as of the lifestyle risk factors, such as smoking and alcohol intake. All parameters were statistically compared between the two cohorts using appropriate statistical tests. Statistical significance was set at *p* < 0.05 and significant differences are indicated in bold font. ^1^ Mean (Range); ^a^ Independent-Samples T-test, ^b^ Pearson Chi-Squared (2-sided *p*).

Variable	OSCC Patients (*n* = 31)				Controls (*n* = 31)				*p*-Value
Age (years) ^1^	69.9 (51–80)				66.7 (53–72)				0.224 ^a^
Gender *n* (%)	Male		Female		Male		Female		0.798 ^b^
	17 (54.8%)		14 (45.2%)		18 (58.1%)		13 (41.9%)		
Smoking *n* (%)	Smokers		Non-Smokers		Smokers		Non-Smokers		**0.011** ^b^
	20 (64.5%)		11 (35.5%)		10 (32.3%)		21 (67.7%)		
Smoking Status *n* (%)	None	Light	Moderate	Heavy	None	Light	Moderate	Heavy	**0.034** ^b^
	11 (35.5%)	7 (22.6%)	7 (22.6%)	6 (19.4%)	21 (67.7%)	6 (19.4%)	1 (3.2%)	3 (9.7%)	
Alcohol Consumption *n* (%)	Yes		No		Yes		No		0.799 ^b^
	16 (51.6%)		15 (48.4%)		15 (48.4%)		16 (51.6%)		
Drinking Status *n* (%)	None	Light	Moderate	Heavy	None	Light	Moderate	Heavy	**0.005** ^b^
	15 (48.4%)	3 (9.7%)	5 (16.1%)	8 (25.8%)	16 (51.6%)	11 (35.5%)	4 (12.9%)	0 (0.0%)	

**Table 4:** Overview of the clinicopathological characteristics of the studied OSCC group, including the anatomical locations (intraoral/lip) and the exact sites of the primary OSCC lesions, the tumor differentiation grade, and the clinical stage (I–IV) of the patients, according to the TNM system.

Primary Tumor Site (*n*, %)					
Intraoral OSCC			Lip OSCC		
25 (80.6%)			6 (19.4%)		
Tongue	Buccal Mucosa	Alveolar Mucosa/Palate	Lip	Floor of Mouth	Retromolar Trigone
6 (19.4%)	4 (12.9%)	7 (22.6%)	6 (19.4%)	4 (12.9%)	4 (12.9%)
OSCC Histological Grade (*n*, %)					
Well Differentiated		Moderately Differentiated		Poorly Differentiated	
6 (19.4%)		20 (64.5%)		5 (16.1%)	
Clinical Stage (TNM-Based) (*n*, %)					
Early-Stage OSCC (I/II)			Late-Stage OSCC (III/IV)		
17 (54.8%)			14 (45.2%)		
Stage I		Stage II	Stage III		Stage IV
8 (25.8%)		9 (29.0%)	6 (19.4%)		8 (25.8%)

**Table 5:** Comparative overview of the target scores (panel coverage %) of each studied miRNA across both bioinformatic analyses, along with the percentage of its observed downregulation in the saliva of OSCC patients compared to controls and the corresponding *p*-values. Statistical significance was set at *p* < 0.05, and significant differences are indicated in bold font. Log_2_FC values denote the log_2_-transformed fold change (FC) in expression levels between OSCC patients and controls and are included as a complement to enable a standardized comparison of the expression differences. ^a^ Mann–Whitney U test. * Although miR-484 emerged solely from the stage-specific analysis, its corresponding target score in the disease-level panel (46.7%) is additionally presented to enable the calculation of the mean target score, reported in this table.

Identified miRNA	Disease-Level Target Score (*n*/15, %)	Stage-Specific Target Score (*n*/6, %)	Mean Target Score (%)	OSCC Downregulation (%, Log_2_FC)	*p*-Value ^a^
			66.7%	67.1% (−1.60)	***p* < 0.001**
miR-181d-5p	10/15 (66.7%)	4/6 (66.7%)			
		
			65%	48.9% (−0.97)	***p* < 0.001**
miR-484	7/15 (46.7%) *	5/6 (83.3%)			
		
			75%	48.7% (−0.96)	***p* = 0.008**
miR-185-5p	10/15 (66.7%)	5/6 (83.3%)			
		
			96.7%	27.4% (−0.46)	***p* = 0.026**
miR-20b-5p	14/15 (93.3%)	6/6 (100.0%)			
		

## Data Availability

The original data presented in this research will be available upon request to the corresponding authors. Certain data may be subject to limited availability in accordance with confidentiality and privacy-protecting protocols or ongoing study conduction.
